# Contralesional Trunk Rotation Dissociates Real vs. Pseudo-Visual Field Defects due to Visual Neglect in Stroke Patients

**DOI:** 10.3389/fneur.2017.00411

**Published:** 2017-08-17

**Authors:** Thomas Nyffeler, Rebecca E. Paladini, Simone Hopfner, Oliver Job, Tobias Nef, Tobias Pflugshaupt, Tim Vanbellingen, Stephan Bohlhalter, René M. Müri, Georg Kerkhoff, Dario Cazzoli

**Affiliations:** ^1^Perception and Eye Movement Laboratory, Departments of Neurology and Clinical Research, Inselspital, Bern University Hospital and University of Bern, Bern, Switzerland; ^2^Gerontechnology and Rehabilitation Group, University of Bern, Bern, Switzerland; ^3^Neurology and Neurorehabilitation Center, Luzerner Kantonsspital, Luzern, Switzerland; ^4^Eye Clinic, Luzerner Kantonsspital, Luzern, Switzerland; ^5^ARTORG Center for Biomedical Engineering Research, University of Bern, Bern, Switzerland; ^6^University Neurorehabilitation Clinics, Department of Neurology, Inselspital, Bern University Hospital and University of Bern, Bern, Switzerland; ^7^Clinical Neuropsychology and Neuropsychological Outpatient Unit, Saarland University, Saarbrücken, Germany

**Keywords:** visual field defect, visual neglect, visual attention, Goldmann perimetry, stroke

## Abstract

In stroke patients, the clinical presentation of visual field defects (VFDs) is frequently accompanied by visual neglect, i.e., the inability to attend and respond to the contralesional space. However, the diagnostic discrimination between the lack of reactions to contralesional stimuli due to VFDs or visual neglect is challenging during clinical examination. This discrimination is particularly relevant, since both clinical pictures are associated with different therapeutic approaches and outcomes. The aim of this study was to systematically investigate the effectiveness of trunk rotation toward the contralesional side—a manipulation dissociating the coordinate system of the trunk from that of the head and eyes—in disentangling real VFDs from “pseudo-VFDs” that occur due to visual neglect. Twenty patients with a left-sided VFD after a right-hemispheric stroke (10 additionally showing visual neglect in neuropsychological testing, VFD + neglect; 10 without neglect, VFD) were tested with Goldmann perimetry in both standard and trunk rotation conditions. In the standard condition, both VFD and VFD + neglect patients showed a conspicuous narrowing of the left visual field. However, trunk rotation triggered strikingly different patterns of change in the two groups: it elicited a significant increase in visual field extension in the VFD + neglect group, but left visual field extension virtually unchanged in the VFD group. Our results highlight contralesional trunk rotation as a simple, viable manipulation to effectively and rapidly disentangle real VFDs from “pseudo-VFDs” (i.e., due to visual neglect) during clinical examination.

## Introduction

Visual field defects (VFDs) are common in stroke patients. For instance, 73% of patients with an infarction within the territory of the middle cerebral artery suffer from hemianopia (1). The diagnosis of VFDs is important, since patients with VFDs are significantly less independent than patients with an intact visual field, and they are significantly impaired in the activities of daily living (2, 3). In some instances, stroke patients not only have VFDs but also present with combined visual neglect. Neglect is an attentional syndrome defined as the failure to detect, respond, or orient to stimuli located in the portion of space contralateral to a brain lesion (4). Similar to VFDs, neglect is common, occurring in up to 43% of patients in the acute phase after a right-hemispheric stroke and receding to 17% after 3 months (5). Neglect is also an independent predictor of poor outcome, in terms of poststroke functional independence (6, 7). Patients suffering from VFDs with additional visual neglect are known to be more impaired than patients suffering from VFDs alone (i.e., without visual neglect) (8–10).

During bedside neurological examination, the diagnostic discrimination between VFDs and visual neglect is particularly challenging. The confrontation method, during which the examiner is facing the patient and is comparing the patient’s visual field with his or her own, is commonly used. In this assessment, both patients with VFDs and patients with visual neglect may not report stimuli presented in the contralesional field. In other words, the conventional confrontation method is a valuable screening to assess whether a patient presents with a disturbance of visual perception; however, ascertaining whether this disturbance is due to a VFD and/or to visual neglect may often not be possible with this method (9, 11, 12). Similarly, during more sophisticated assessments such as Goldmann perimetry, it is difficult to attribute the lack of reactions to contralesional stimuli to VFDs or to visual neglect.

The aim of this study was to assess the efficiency of a diagnostic procedure that may help clinicians to disentangle “real” VFDs (i.e., without visual neglect) from “pseudo-VFDs” that are due to impaired attention. The boundaries of VFDs are defined in a retinal coordinate system (13–15). For visual neglect, the boundaries of the neglected space can be defined according to different reference frames, a main distinction being represented by allocentric or object-centered neglect (in which the spatial coordinates system is centered around external objects, regardless of their position in space with respect to the viewer) and by egocentric or viewer-centered neglect (in which the spatial coordinates system is centered around the viewer) [e.g., (16)]. For egocentric visual neglect, previous studies have shown that the boundaries of the unattended space are defined with respect to the head (17, 18) and, more crucially, to the midline of the trunk (19–21). This suggests that, in neglect patients, visual perception can be modulated when the coordinate system centered around the head or the trunk is dissociated from the retinotopic coordinate system. Indeed, Kooistra and Heilman (22) described the case of a single patient with left-sided visual neglect who could report more easily the presence of moving fingers in the left visual field when his gaze was directed toward the right. Similarly, Vuilleumier et al. (18) described the cases of two neglect patients whose left visual field improved when the eyes were looking to the right. These few previous reports thus provide anecdotal evidence that a dissociation of the coordinate systems may be an elegant procedure to disentangle the effects of VFDs from the ones of visual neglect. However, the efficacy and specificity of this diagnostic procedure have never been systematically evaluated in a larger sample of patients. One reason for this lack of systematic investigations might be that a dissociation of the coordinate systems, defined with respect to the retina and head, is not practicable in static or kinetic visual field perimetry, which is considered the gold standard for visual field assessment. In fact, during perimetry, the midlines of both the visual field and the head are parallel and oriented straight toward the middle of the projection screen.

Another approach to dissociate coordinate systems during perimetry, which seems to be technically more viable, may be to rotate the trunk axis away from the axes of the eyes and of the head. Contralesional trunk rotation has indeed been shown to ameliorate visual perception in single cases of patients with visual neglect (23–25). In other words, rotating the trunk toward the left, contralesional space (with respect to the eyes and the head) is able to reduce the portion of neglected space (and thus increase the portion of space in which patients are able to respond to visual stimuli), because the critical pivot of the spatial reference system for neglect (i.e., the trunk midline) is rotated toward the same side. In contrast, the leftward rotation of the trunk (with respect to the eyes and the head) has no influence on the lack of responses due to “real VFDs” (i.e., due to damage to the visual system), because the critical pivot of the spatial reference system for VFDs (i.e., the midline of the retina) is not rotated. Thus, trunk rotation can hypothetically be applied in patients suffering from a contralesional impairment of visual perception resulting from VFDs and/or neglect to test whether a dissociation of the coordinate system of the trunk from the one of the eyes and of the head might help to disentangle “real VFDs” (i.e., resulting from damage to the visual system) from “pseudo-VFDs” (i.e., resulting from damage to the visual attentional system).

We systematically assessed 20 right-hemispheric stroke patients, who presented left-sided VFDs during clinical beside examination by the aforementioned confrontation method. Among these 20 patients, 10 additionally presented neglect symptoms in standard neuropsychological testing, whereas 10 did not. All patients were assessed by means of Goldmann perimetry under two conditions: (1) in the standard condition, where the midlines of the visual field, the head, and the trunk were parallel and oriented straight toward the middle of the projection screen; and (2) in the trunk rotation condition, where the midline of the trunk was rotated 30° toward the left, while the midline of the visual field and the head were parallel and oriented straight toward the middle of the projection screen. We hypothesized that trunk rotation toward the left would increase the magnitude of the area in which patients with visual neglect would respond to visual stimuli, i.e., appear as an “extension” of the visual field in the perimetry results. Conversely, in stroke patients without visual neglect, trunk rotation should have no significant effect on the magnitude of the area in which patients would respond to visual stimuli, since the visual field is defined in retinal coordinates.

## Materials and Methods

### Participants

Twenty right-hemispheric stroke patients (aged between 23 and 83 years, mean = 58.3, SD = 17.5; 9 women) participated in the study. All patients had a left VFD, as assessed by means of the clinical bedside confrontation method. Several variants of the confrontation method exist; we applied the “traditional” confrontation method, as described by Elliot et al. (26). In brief, the patient and the examiner face each other, seating at eye level, at a distance of approximately 70 cm, and with their mid-sagittal planes aligned. The background behind the examiner is uniform. Each eye is examined separately, i.e., both the examiner and the patient cover one opposing eye with their hand palm. The patient is asked to maintain a steady fixation on the eye of the examiner; the compliance with this instruction is constantly monitored by the examiner. The examiner then introduces a finger in the visual field as a target, moving it from the periphery to the center, along a spatial plane that is equidistant between examiner and patient. The patient is asked to indicate when he or she first sees the finger; the indicated location is compared with the one seen by the examiner. The different portions of the visual field are systematically tested.

Ten of the 20 patients additionally had visual neglect, as assessed by means of a cancellation test [the Bells Test (27), the Random Shape Cancellation Test (28), or the Star Cancellation Test (29)] and the Line Bisection Test (30) (Table 1). To be considered as presenting with neglect, the patients had to show clinically relevant scores in at least one of the two tests. The mean interval between stroke onset and testing was 159 days (SD = 322.6, range 10–1,355 days). There was no statistically significant difference between the two groups (patients with or without neglect) in terms of the mean interval between stroke onset and testing [*t*(18) = −1.59, *p* = 0.129, two-tailed]. All participants had normal or corrected-to-normal visual acuity.

**Table 1:** Sociodemographic and clinical characteristics of patients with visual field defects (VFD) and with VFD + neglect.

No.	Group	Age range (years)	Etiology	Time since onset (days)	CoC^a^	Line bisection (%)^b^
1	VFD + neglect	56–60	Ischemic	10	0.807^c^	41.0^c^
2	VFD + neglect	76–80	Ischemic	28	0.936^c^	43.4^c^
3	VFD + neglect	66–70	Ischemic	36	0.256^c^	78.5^c^
4	VFD + neglect	81–85	Ischemic	39	0.764^c^	3.9
5	VFD + neglect	61–65	Ischemic	45	0.632^c^	10.4
6	VFD + neglect	66–70	Ischemic	46	0.838^c^	81.4^c^
7	VFD + neglect	81–85	Ischemic	48	0.137^c^	10.3
8	VFD + neglect	71–75	Hemorrhagic	58	0.614^c^	10.0
9	VFD + neglect	46–50	Hemorrhagic	75	0.093^c^	9.8
10	VFD + neglect	76–80	Ischemic	79	0.215^c^	37.0^c^
11	VFD	36–40	Hemorrhagic	13	0.022	0.69
12	VFD	21–25	Hemorrhagic	28	0	−5.71
13	VFD	56–60	Hemorrhagic	37	0.015	2.76
14	VFD	61–65	Hemorrhagic	40	−0.018	5.92
15	VFD	56–60	Ischemic	46	0	−0.31
16	VFD	36–40	Ischemic	60	0	−1.9
17	VFD	51–55	Ischemic	154	0	2.34
18	VFD	31–35	Hemorrhagic	180	0	−10.63
19	VFD	25–30	Ischemic	815	0	−4.96
20	VFD	71–75	Ischemic	1,355	0	2.4

*^a^Center of Cancellation, based on the Bells Test (27), the Random Shape Cancellation Test (28), or the Star Cancellation Test (29). The CoC was calculated with the freely available software cancel.exe, developed and validated by Rorden and Karnath (31)*.

*^b^The mean percentage of the rightward deviation—relative to the true half of lines—is reported. Positive values indicate rightward shifts and negative values indicate leftward shifts*.

*^c^Clinically relevant test score, i.e., above the cut-off value of 0.08 for the CoC in the cancellation tests (31) and of 11% in the Line bisection test (32)*.

**Figure 1:**
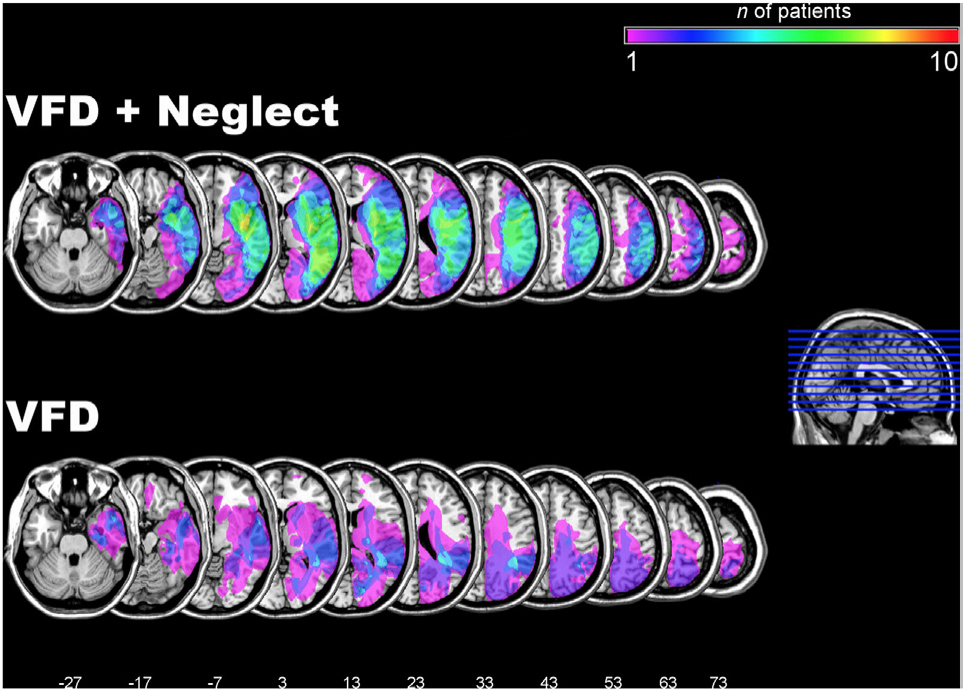
Lesion overlap maps in the two groups of patients. Lesion overlap maps in the group of patients with visual field defects (VFDs) and additional neglect, as assessed by neuropsychological testing (VFD + neglect; upper row) and in the group with VFDs but no neglect (VFD; lower row). The color-coded legend at the top right of the figure depicts the number of patients in each group with damage to a specific brain region. The overlap maps are plotted onto axial slices of the ch2 template of the Montreal Neurological Institute (MNI) brain. The axial slices are oriented according to the neurological convention and are depicted in ascending steps of 10 mm. The z-position of each axial slice in the MNI Talairach stereotaxic space is indicated by the numbers at the bottom of the figure and also depicted by the blue lines on the sagittal slice on the right hand of the figure.

To depict the localization of the brain lesions and their overlap in the two groups of patients, we computed lesion overlap maps. To this aim, the lesions of the patients were mapped on their individual, structural MRI images by means of the MRIcron software (33). We adopted the same procedure outlined by Karnath et al. (34, 35), i.e., diffusion-weighted scans were used for lesion mapping if an MRI was conducted within the first 48 h poststroke, otherwise T2-weighted scans were used. The borders of the lesions were directly delineated on every transverse slice of the individual MRI images. The MRI images and the lesion volumes were then mapped into approximate Montreal Neurological Institute space, by means of the spatial normalization algorithm provided by the SPM12 software (http://www.fil.ion.ucl.ac.uk/spm/). In the few cases in which only CT scans were available, lesions were also directly delineated on every transverse slice of the individual CT images and then spatially normalized by means of the algorithm provided by the SPM clinical toolbox (36). Finally, lesion overlap maps for the two groups of patients were computed and depicted by means of the MRIcron software (33) and are presented in Figure 1.

### Experimental Procedures

The left visual field of the left eye was assessed using a standard Goldmann visual field perimeter (Haag-Streit Diagnostics, Bern, Switzerland). The left eye was chosen since the left temporal visual field of the left eye is larger than the left nasal visual field of the right eye. The perimeter was calibrated to a luminosity of 1000 lx in the test sphere, and the test stimuli were then adjusted accordingly in a standard manner. To ensure the same experimental conditions across all patients, the stimulus size V/4 was applied. In the standard condition, the midlines of the visual field, the head, and the trunk were kept strictly parallel and oriented straight toward the middle of the projection screen. In the trunk rotation condition, the midline of the trunk was rotated 30° toward the left side, whereas the midlines of the visual field and of the head were parallel and oriented straight toward the middle. The starting locations of the moving stimuli were selected randomly for each trial. The order standard condition/trunk rotation condition was counterbalanced across participants. Central visual fixation was monitored throughout the whole perimetry, using the built-in telescope.

### Data Analysis

In a first step, the isopters obtained from the standard condition (standard perimetry) and from the trunk rotation condition were measured, in degrees, for each patient. For the purposes of statistical analysis, to quantify the extension of the left visual field by means of a single value for each patient and each condition (i.e., standard condition, trunk rotation condition), the following procedure was applied: (1) the extension of the left visual field was quantified in terms of degrees of eccentricity within 12 radial sectors of 15° each, defined as the space between two radial meridians (i.e., 90°–105°, 105°–120°, 120°–135°, and so forth, up to 255°–270°); this resulted in 12 values per patient and per condition; (2) the values corresponding to the two radial sectors adjacent to the vertical meridian (i.e., 90°–105° and 255°–270°) were excluded from analysis; this was due to the fact that these two sectors are close to the midline, at the very border with the right visual field, and thus cannot be univocally attributed to the left visual field; this resulted in 10 values per patient and per condition; (3) finally, the 10 values of each condition were averaged within every patient, resulting in 1 value per patient and per condition; this value is henceforth referred to as “mean left visual field extension.”

To assess possible changes in the extension of the left visual field after trunk rotation, we analyzed the values obtained with the abovementioned procedure by means of a mixed-design analysis of variance with two factors: (1) condition (levels: standard condition, trunk rotation condition; within-subjects factor) and (2) group (levels: patients with VFD + neglect; patients with VFD; between-subjects factor). All subsequent *post hoc* analyses were conducted using Bonferroni-corrected *t*-tests, and the alpha level was set at *p* < 0.05.

## Results

**Figure 2:**
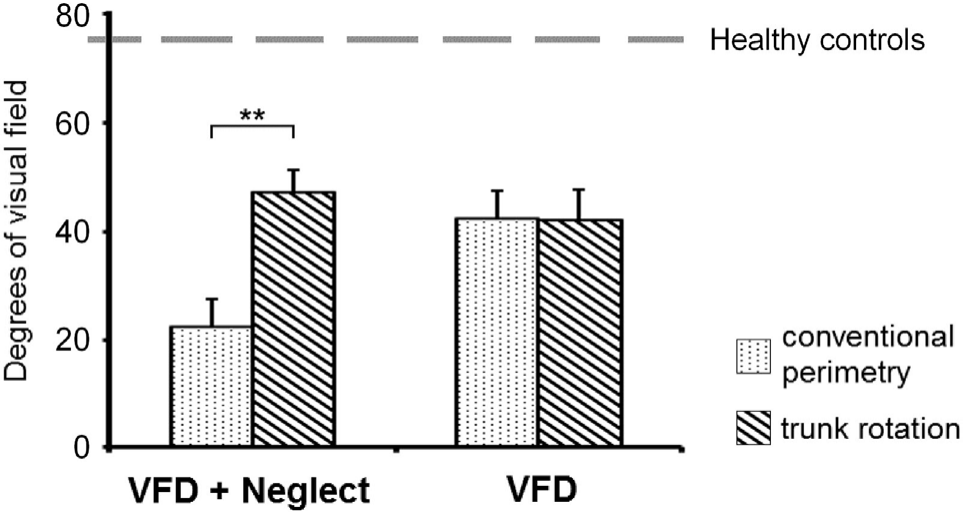
Numeric representation of the mean left visual field extension. Mean left visual field extension (calculated as the mean degrees of eccentricity within radial sectors of 15° each, defined as the space between two radial meridians; the values corresponding to the two radial sectors adjacent to the vertical meridian were excluded from analysis due to their adjacency to the midline and the right visual field; see the Section “Materials and Methods” for a detailed description), as obtained by means of conventional perimetry (dotted bars) and by means of perimetry during contralesional trunk rotation (striped bars), in the group of patients with visual field defects (VFDs) and additional neglect, as assessed by neuropsychological testing (VFD + neglect; left hand side), and in the group with VFDs but no neglect (VFD; right hand side). Please note that, after trunk rotation (eliminating the neglect component), the visual field extension in the VFD + neglect group (which was initially much smaller, i.e., 22°), became similar to the one of the VFD group (i.e., 47° and 42° respectively). The gray, dashed horizontal line represents the normal mean value of the isopter as measured in healthy individuals (75°) according to Niederhauser and Mojon (37). Error bars depict the SEM. Asterisks denote significant *post hoc* tests (**p < 0.001).

In all stroke patients, conventional perimetry (standard condition) revealed a left-sided VFD. In patients with VFD + neglect, the mean left visual field extension was of 22°, whereas in patients with VFD, this extension was of 42°. The results of the perimetry in the trunk rotation condition showed that the left visual field extension significantly increased in patients with VFD + neglect, but remained unchanged in patients with VFD. The statistical analysis of the left visual field extension revealed a significant main effect of the condition [i.e., standard condition vs. trunk rotation condition; *F*(1, 18) = 42.612, *p* < 0.001] and a non-significant main effect of the group [i.e., VFD + neglect vs. VFD; *F*(1, 18) = 1.003, *p* = 0.330]. Crucially, the analysis yielded a highly significant interaction between the condition and the group [*F*(1, 18) = 44.378, *p* < 0.001]. Patients with VFD + neglect showed a significant increase in the mean left visual field extension when their trunk was rotated 30° toward the left (mean left visual field extension in the trunk rotation condition = 47°). In contrast, in patients with VFD, trunk rotation did not significantly alter the left visual field extension (mean left visual field extension in the trunk rotation condition = 42°). The results concerning this interaction and the corresponding *post hoc* tests are depicted in Figure 2.

**Figure 3:**
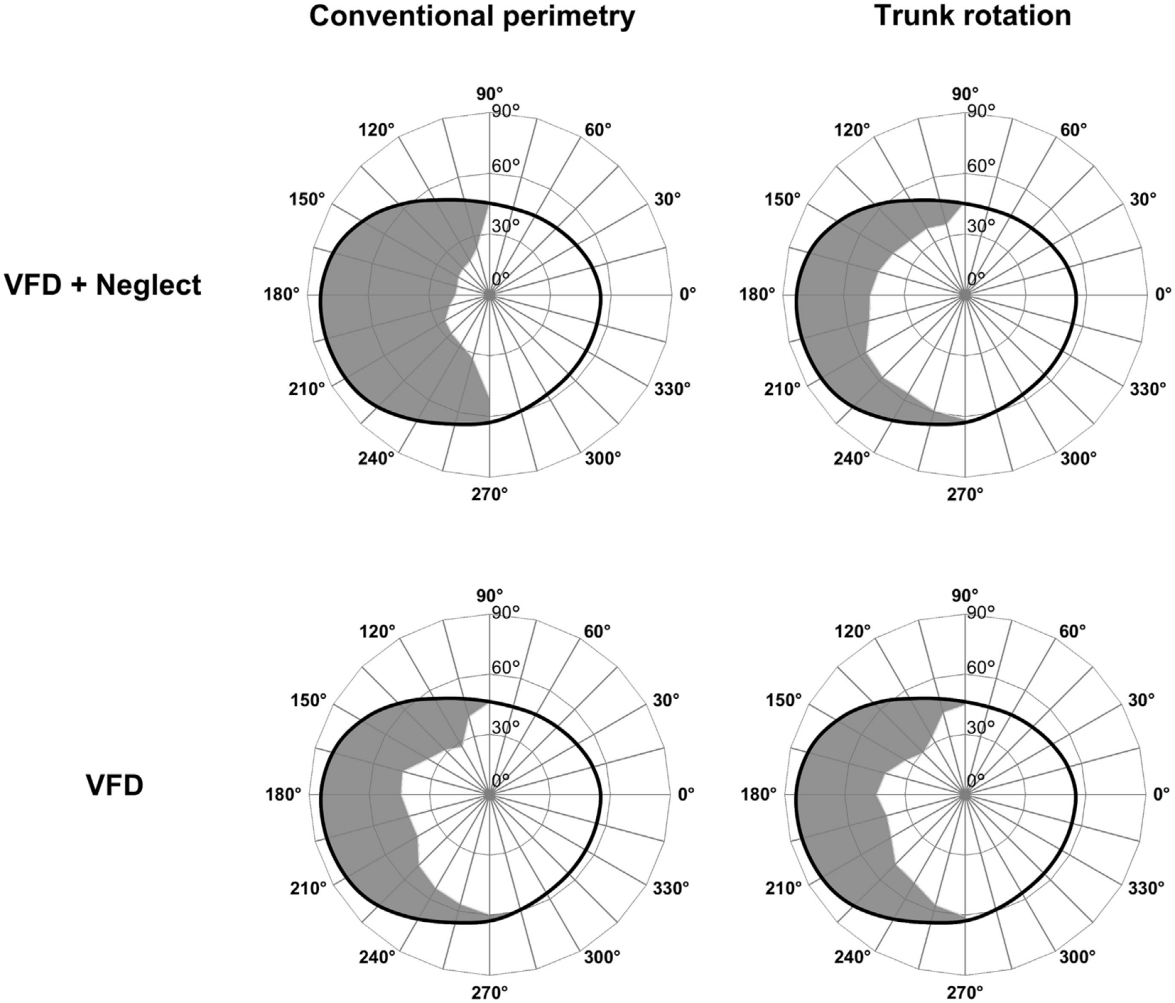
Graphic representation of the mean left visual field extension. Mean left visual field extension as obtained by means of conventional perimetry (left column) and by means of perimetry during contralesional trunk rotation (right column), in the group of patients with visual field defects (VFDs) and additional neglect, as assessed by neuropsychological testing (VFD + neglect; top row), and in the group with VFDs but no neglect (VFD; bottom row). The gray-colored surfaces represent the portions of the left visual field in which the patients gave no answer (i.e., they did not acknowledge the presence of a visual stimulus). The black, ovaloid lines represent the normal mean isopter as measured in healthy individuals, according to Niederhauser and Mojon (37).

**Figure 4:**
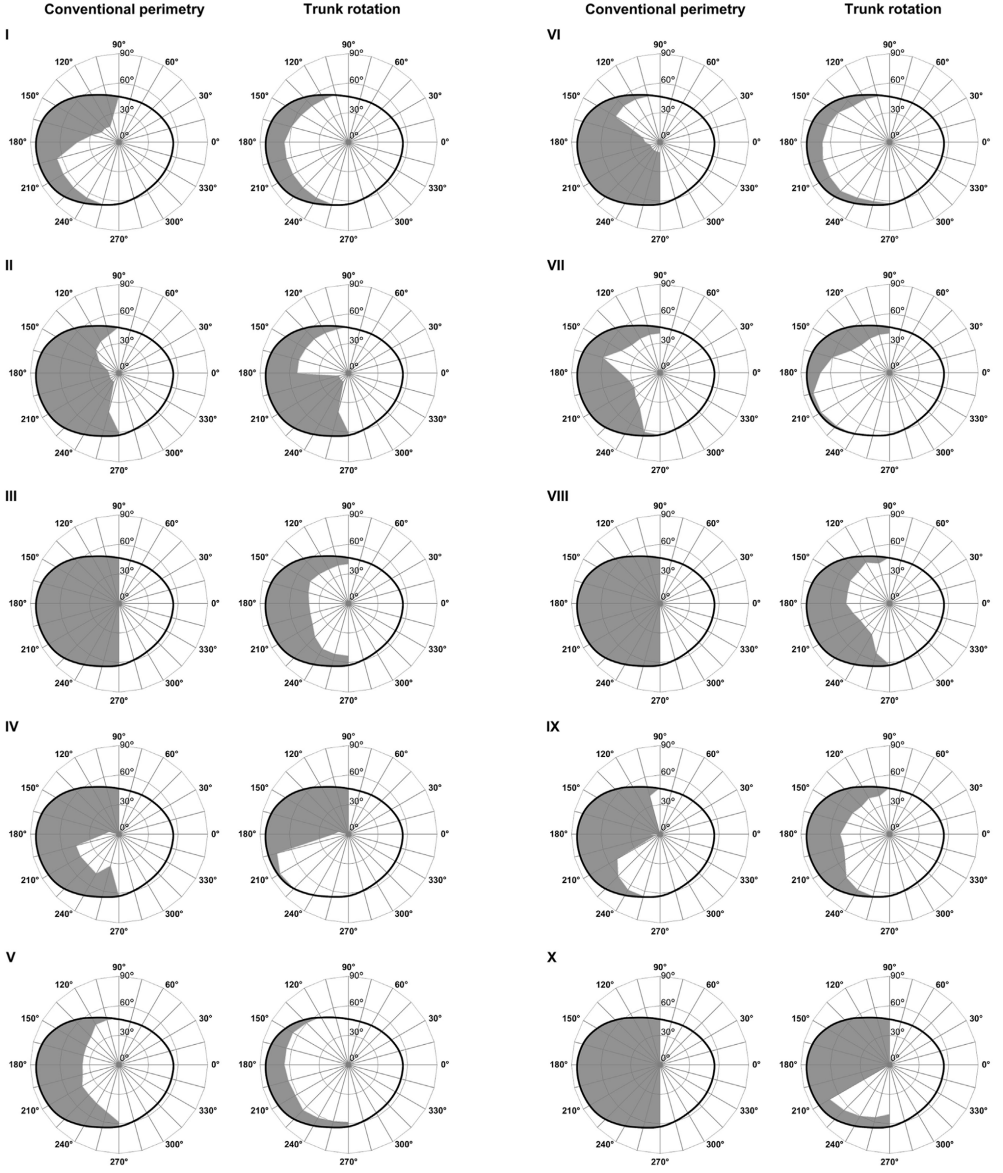
Individual left visual fields in visual field defect (VFD) + neglect patients. Individual left visual fields of the 10 patients with left VFDs and additional left neglect, as assessed by neuropsychological testing (VFD + neglect; numbered I–X), measured by means of conventional perimetry (left columns) and perimetry during contralesional trunk rotation (right columns). The gray-colored surfaces represent the portions of the left visual field in which the patients gave no answer (i.e., they did not acknowledge the presence of a visual stimulus). The black, ovaloid lines represent the normal mean isopter as measured in healthy individuals, according to Niederhauser and Mojon (37). Note that perimetry during the trunk rotation condition triggered a conspicuous increase of the visual field extension in all patients.

To further illustrate these effects, the mean left visual field extension in the group with VFD + neglect and in the group with VFD is depicted in Figure 3. Moreover, to illustrate the significant effects of trunk rotation in patients with VFD + neglect on an individual level, single cases are shown in Figure 4. In this figure, it can also be observed that trunk rotation increased the visual field extension not only in its central portions, but also in the periphery (see, for example, patient V and patient VII).

## Discussion

The results of this study show that a trunk rotation of 30° during visual field testing is a reliable procedure to disentangle “pseudo-VFDs” (i.e., resulting from damage to the visual attention system), from “real VFDs” (i.e., resulting from damage to the visual system). In 20 right-hemispheric stroke patients, who presented with left VFDs as assessed by the bedside confrontation method, standard Goldmann perimetry, performed under dissociation of the trunk axis from the axes of the eyes and of the head, showed a significant decrease of the left-sided “pseudo-VFD” in patients who additionally suffered from visual neglect. The same procedure, however, had no significant effect in patients without neglect.

The diagnostic differentiation between VFDs and visual neglect is relevant, since both the outcomes and the therapeutic approaches for these two disorders are substantially different (2, 9, 38, 39). In some patients participating in our study, the results of the clinical bedside confrontation method and of the standard Goldmann perimetry suggested a complete left-sided hemianopia. The trunk rotation procedure, however, revealed that these test results were confounded by the presence of visual neglect and that the visual system *per se* was intact. In these patients, trunk rotation reduced the negative effects of visual neglect. For instance, what appeared to be a dense hemianopia according to standard testing, was reverted to a quadrantanopia under trunk rotation conditions, disentangling defects due to visual neglect and to VFDs.

These results are due to the fact that the boundaries of VFDs are coded in a retinal coordinate system (13–15), whereas the boundaries of the neglected space in egocentric visual neglect are coded with respect to the position of the midline of the head and, more crucially, of the trunk (17–19, 21). Hence, a rotation of the trunk can modulate the extension of the visual field in which the patient is not able to respond to visual stimuli due to visual neglect, but will not affect the extension of the visual field in which the patient is not able to respond to visual stimuli due to a VFD. In conventional circumstances, in which the coordinates of the retina, the head, and the trunk are aligned, the lack of responses in a given portion of the visual field cannot thus be univocally attributed to neglect or to a VFD. The contralesional rotation of the trunk allows to dissociate the coordinates systems (i.e., keep retinal and head coordinates aligned, while displacing trunk coordinates) and to assess whether the absence of responses in a given part of the visual field is due—at least in part—to visual neglect.

Our results nicely correspond with findings from electrophysiological studies. The latencies of visual evoked potentials, obtained in four patients with left-sided neglect, have been shown to be significantly longer in the left than in the right visual field when patients were tested in a standard condition (i.e., with head and trunk oriented straight ahead with respect to the screen) (40, 41). However, when the trunk of the patients was rotated toward the left, latencies became comparable across both hemifields (41). Similarly, our results are in line with findings obtained in three patients with left-sided neglect where stimuli were tachistoscopically presented in the left or right visual half-field. In this study, trunk rotation to the left improved neglect patients’ visual perception, as compared to a normal upright position (i.e., with the trunk, head, and gaze oriented straight ahead) (24).

Some patients with left-sided neglect show a strong “magnetic attraction” toward the right side of space, i.e., they repeatedly return with their gaze to the same right-sided objects (42). In these patients, the bedside confrontation method can be made more reliable by positioning the patient with his or her right, intact side close to a wall, thus forcing the visual attention more toward the left, contralesional side (11). Although very interesting from a clinical point of view, this procedure is not viable in the context of standard field plotting techniques, such as during Goldmann perimetry.

In conclusion, this study shows that a trunk rotation of 30° toward the contralesional side is a viable and useful procedure to substantially help in the diagnostic distinction between “real VFDs” and “pseudo-VFDs” in stroke patients during perimetry. Importantly, the clear-cut results of our systematic examination, obtained by means of perimetry, strongly suggest that trunk rotation could also be easily and rapidly applied during bedside examination, in which visual field testing is often performed by means of finger perimetry with the confrontation method. This is very relevant because (a) as shown, the extension of a VFD could be overestimated due to the confounding effect of visual neglect; and (b) an accurate distinction between “real VFDs” and “pseudo-VFDs” is of particular interest for advising patients and their relatives regarding stroke outcome and therapy planning.

## Ethics Statement

This study was carried out in accordance with the recommendations of the latest version of the Declaration of Helsinki. All subjects gave written informed consent. The protocol was approved by the Ethics Committees of the States of Bern and Lucerne.

## Author Contributions

ThN, TP, OJ, TV, SB, RM, GK, and DC designed the study; ThN and SH collected data; ThN, RP, SH, ToN, TP, and DC analyzed the data; ThN, RP, SH, GK, and DC wrote the manuscript; and ThN, RP, SH, OJ, ToN, TP, TV, SB, RM, GK, and DC reviewed the manuscript.

## Conflict of Interest Statement

The authors declare that the research was conducted in the absence of any commercial or financial relationships that could be construed as a potential conflict of interest.
